# Weekly Cisplatin Cycles and Outcomes for Chemoradiation in Head and Neck Cancer

**DOI:** 10.1001/jamanetworkopen.2024.50272

**Published:** 2024-12-09

**Authors:** Sung Jun Ma, Simeng Zhu, Jas Virk, Andrew Koempel, Priyanka Bhateja, Emile Gogineni, Sujith Baliga, David Konieczkowski, Darrion Mitchell, Sachin Jhawar, John Grecula, Matthew Old, James Rocco, Marcelo Bonomi, Dukagjin Blakaj

**Affiliations:** 1 Department of Radiation Oncology, The Arthur G. James Cancer Hospital and Richard J. Solove Research Institute, The Ohio State University Comprehensive Cancer Center, Columbus; 2 Jacobs School of Medicine and Biomedical Sciences, University at Buffalo, The State University of New York, Buffalo; 3 Department of Medical Oncology, The Arthur G. James Cancer Hospital and Richard J. Solove Research Institute, The Ohio State University Comprehensive Cancer Center, Columbus; 4 Department of Otolaryngology, The Arthur G. James Cancer Hospital and Richard J. Solove Research Institute, The Ohio State University Comprehensive Cancer Center, Columbus

## Abstract

**Importance:**

National Comprehensive Cancer Network guidelines recommend weekly cisplatin as an alternative concurrent systemic therapy for definitive chemoradiation in patients with head and neck cancer. However, the impact of different levels of adherence to weekly cisplatin on outcomes stratified by human papillomavirus p16 status remains unclear.

**Objective:**

To evaluate the association between the number of weekly cisplatin cycles and outcomes.

**Design, Setting, and Participants:**

This retrospective, observational, single-institution cohort study at The Ohio State Comprehensive Cancer Center included patients with a diagnosis of nonmetastatic head and neck cancer between December 1, 2011, and March 30, 2020, who received chemoradiation. Data analysis was performed between March and May 2024.

**Exposure:**

A total of 5, 6, or 7 to 8 weekly cisplatin cycles.

**Main Outcomes and Measures:**

The primary outcomes were overall survival (OS), progression-free survival (PFS), locoregional failure (LRF), and distant failure (DF). Cox multivariable analysis was performed for variables associated with OS and PFS, and Fine-Gray multivariable analysis was performed for variables associated with LRF and DF.

**Results:**

A total of 142 patients met the criteria (119 men [83.8%]; median [IQR] age, 59 [54-63] years). Median (IQR) follow-up was 46.8 (40.8-55.6) months. Among 92 patients with reasons for cisplatin interruption reported, the most common reason was low blood counts (42 patients [45.7%]). Those who missed weekly cisplatin cycles had worse OS (adjusted hazard ratio [aHR], 2.22; 95% CI, 1.19-4.17; *P* = .01) and PFS (aHR, 1.83; 95% CI, 1.06-3.15; *P* = .03) than those who received 7 to 8 cycles. Cancer control outcomes were comparable between these groups (LRF aHR, 0.53; 95% CI, 0.15-1.93; *P* = .34; DF aHR, 1.51; 95% CI, 0.60-3.82; *P* = .38). Patients with p16-negative tumors who missed weekly cisplatin cycles had worse OS (for every missing cisplatin cycle, aHR, 11.34, 1.51-84.94; *P* = .02) than those treated with 7 to 8 cycles. However, for those with p16-positive tumors, there were no statistically significant differences in OS between those who missed weekly cisplatin cycles vs others who received 7 to 8 cycles (aHR, 1.21; 95% CI, 0.47-3.14; *P* = .69).

**Conclusions and Relevance:**

In this cohort study of patients with head and neck cancer who received definitive chemoradiation, those with p16-negative tumors who missed weekly cisplatin cycles had lower OS than those who received 7 to 8 cycles, although OS was comparable between these groups for p16-positive tumors. Cytopenia represented the most common reason for cisplatin interruption.

## Key Points

**Question:**

What is the association of different levels of adherence to weekly cisplatin with survival and cancer control outcomes among patients with head and neck cancer who received chemoradiation?

**Findings:**

In this cohort study involving 142 patients, those who missed cisplatin cycles had lower survival outcomes than those who received 7 to 8 cycles, but oncologic outcomes were comparable. This finding was also seen among patients with p16-negative tumors.

**Meaning:**

These findings suggest that missing several cycles of weekly cisplatin is associated with worse survival, even among those with p16-negative tumors; low blood counts were the most common reason for missing chemotherapy.

## Teaser

This cohort study evaluates the association between the number of weekly cisplatin cycles and outcomes in patients receiving chemoradiation treatment for head and neck cancer.

## Introduction

The National Comprehensive Cancer Network guideline recommends high-dose cisplatin delivered every 3 weeks as a category 1, preferred concurrent chemotherapy option for definitive chemoradiation among patients with head and neck cancer.^1^ However, more than one-quarter of patients could not receive the third cycle in a recent meta-analysis, as well as in the RTOG 0129 trial.^2,3^ Given that there were no statistically significant differences in outcomes between receiving 2 vs 3 cycles total, 200 mg/m^2^ was suggested as a minimum cumulative dose to be effective for optimal oncologic outcomes.^3^

As an alternative regimen, weekly cisplatin was also recommended for consideration by the National Comprehensive Cancer Network guideline.^1^ Although both cisplatin schedules have been shown to have comparable locoregional control and survival outcomes in a recent meta-analysis,^2^ the rate of cisplatin interruptions has been shown to be similar regardless of cisplatin schedules. However, there is a paucity of literature regarding the impact of different levels of adherence to weekly cisplatin on outcomes stratified by p16 human papillomavirus status. For instance, the majority of patients included in several recent phase 3 trials of weekly cisplatin had either oral cavity cancer or p16-negative oropharyngeal cancer.^4,5^ To address this knowledge gap, we performed an observational cohort study of patients with head and neck cancer who underwent definitive chemoradiation with weekly cisplatin, evaluating the association between the number of cisplatin cycles and outcomes stratified by p16 status.

## Methods

This cohort study was reviewed and approved by The Ohio State University Comprehensive Cancer Center institutional review board. It follows the Strengthening the Reporting of Observational Studies in Epidemiology (STROBE) reporting guidelines. Given the retrospective nature of our study, a waiver of consent was obtained, in accordance with 45 CFR §46. The consent process was not deemed to be feasible, and obtaining consent retrospectively was thought to pose a greater risk in comparison.

Our institutional database was queried for patients who received a diagnosis of nonmetastatic squamous cell carcinoma of the head and neck between December 2011 and March 2020 who underwent definitive chemoradiation with weekly cisplatin (40 mg/m^2^). All patients in our study underwent intensity modulated radiation therapy with 69.96 to 70 Gy for 33 to 35 fractions total and 5 or more cycles of weekly cisplatin. Patients who underwent surgery, radiation alone, or other concurrent systemic therapy regimens were excluded for analysis. Patients who underwent fewer than 5 weekly cisplatin cycles were also excluded given their small sample size (18 patients [11.3%]).

In addition to the number of weekly cisplatin cycles and reasons for not receiving chemotherapy, other variables of interest included age, gender, race, smoking status, primary disease site, body mass index, T and N category based on the American Joint Committee on Cancer 7th edition, Eastern Cooperative Oncology Group performance status (ECOG PS), and human papillomavirus status. All missing values were coded as unknown for analysis. Patients self-reported their race and ethnicity. For the purposes of this analysis, racial categories were collapsed to White and other, which includes but is not limited to African American, American Indian or Alaska Native, Asian, Hispanic, unknown, and those who declined to answer. These categories were grouped together as a single group, because it would be challenging to demonstrate meaningful differences in outcomes, in part, owing to small subgroup sample sizes. Data on race are included in this study because racial differences may be associated with clinical outcomes.

The primary end points of our study were overall survival (OS) and progression-free survival (PFS). OS was defined as time interval from diagnosis to death from any cause. PFS was defined as time interval from diagnosis to death from any cause or tumor progression. Other end points included locoregional failure (LRF) and distant failure (DF), time intervals from diagnosis to locoregional and distant metastasis, respectively. Tumor progression was determined using a multidisciplinary approach among radiologists, pathologists, surgeons, and medical and radiation oncologists.

### Statistical Analysis

Treatment groups were divided into those who received 5, 6, or 7 to 8 cycles. Weekly cisplatin cycles were evaluated as a continuous variable with 7 to 8 cycles as a reference group. Reasons for missing cisplatin cycles were also recorded. Fisher exact test and Mann-Whitney *U* tests were performed to evaluate baseline characteristics as appropriate. Kaplan-Meier plot and log-rank tests were performed for OS and PFS. Cox multivariable analysis (MVA) was performed for variables associated with OS and PFS. Fine-Gray MVA was performed for variables associated with LRF and DF. To further evaluate differences in outcomes, pairwise comparisons were reported among those who received 5, 6, vs 7 to 8 cycles adjusted for baseline characteristics. As an exploratory, hypothesis-generating analysis, subgroup analysis was performed using Cox MVA among those with p16-positive and p16-negative tumors. Fine-Gray MVA could not be performed on these subgroups, since the number of events was too low for analysis. Two-sided *P* < .05 was considered statistically significant. All statistical analyses were performed using R statistical software version 4.3.2 (R Project for Statistical Computing) from March to May 2024.

## Results

A total of 142 patients met our criteria (119 men [83.8%]; median [IQR] age, 59 [54-63] years). There were 34 patients (24.0%) who received 5 cycles, 58 (40.8%) who received 6 cycles, and 50 (35.2%) who received 7 to 8 cycles (Table 1). Those who received 5 cycles had higher lymph node disease burden, whereas other baseline characteristics were well balanced (Table 1). Median (IQR) follow-up was 46.8 (40.8-55.6) months.

**Table 1.** Baseline Characteristics of Patients by Number of Weekly Cisplatin Cycles

Characteristic	Patients, No. (%)			*P* value
	7-8 Cycles	6 Cycles	5 Cycles	
Age, y				
<65	45 (90.0)	45 (77.6)	25 (73.5)	.10
≥65	5 (10.0)	13 (22.4)	9 (26.5)	
Gender				
Female	7 (14.0)	8 (13.8)	8 (23.5)	.46
Male	43 (86.0)	50 (86.2)	26 (76.5)	
Race				
White	48 (96.0)	52 (89.7)	30 (88.2)	.32
Other^a^	2 (4.0)	6 (10.3)	4 (11.8)	
Smoking				
Never	13 (26.0)	17 (29.3)	12 (35.3)	.82
Former	19 (38.0)	20 (34.5)	14 (41.2)	
Current	16 (32.0)	20 (34.5)	7 (20.6)	
Not available	2 (4.0)	1 (1.7)	1 (2.9)	
Primary site				
Oropharynx	35 (70.0)	47 (81.0)	27 (79.4)	.54
Larynx	11 (22.0)	6 (10.3)	4 (11.8)	
Other	4 (8.0)	5 (8.6)	3 (8.8)	
Body mass index^b^				
Normal (18.5-24.9)	9 (18.0)	15 (25.9)	6 (17.6)	.07
Underweight (<18.5)	2 (4.0)	0 (0.0)	1 (2.9)	
Overweight (25.0-29.9)	11 (22.0)	22 (37.9)	16 (47.1)	
Obese (≥30.0)	28 (56.0)	21 (36.2)	11 (32.4)	
Eastern Cooperative Oncology Group performance status				
0	32 (64.0)	42 (72.4)	28 (82.4)	.20
>0	18 (36.0)	16 (27.6)	6 (17.6)	
T category				
1-2	29 (58.0)	43 (74.1)	22 (64.7)	.21
3-4	21 (42.0)	15 (25.9)	12 (35.3)	
N category				
0-1	23 (46.0)	11 (19.0)	5 (14.7)	.002
2-3	27 (54.0)	47 (81.0)	29 (85.3)	
Human papillomavirus				
Negative	14 (28.0)	10 (17.2)	8 (23.5)	.41
Positive	36 (72.0)	48 (82.8)	26 (76.5)	

^a^ Other includes but is not limited to African American, American Indian or Alaska Native, Asian, Hispanic, and those with unknown race or who declined to answer. These racial categories were grouped together because of the small sample sizes.

^b^ Body mass index is calculated as weight in kilograms divided by height in meters squared.

There were 92 patients (64.8%) who missed cisplatin cycles. Of these patients, 79 patients (85.9%) had 92 reasons documented for lower adherence to weekly cisplatin (Table 2). The top 3 most common reasons were low blood counts (42 patients [45.7%]), failure to thrive (26 patients [28.3%]), and fever or infection (13 patients [14.1%]).

**Table 2.** Reasons for Missing Weekly Cisplatin Cycles

Reasons	Patients, No. (%)
Low blood counts	42 (45.7)
Failure to thrive (nausea, vomiting, dehydration, poor performance status)	26 (28.3)
Fever or infection	13 (14.1)
Acute kidney injury	3 (3.3)
Deep vein thrombosis	1 (1.1)
Rash	1 (1.1)
Chest pain	1 (1.1)
Arrhythmia	1 (1.1)
Other	4 (4.3)

**Figure 1.**
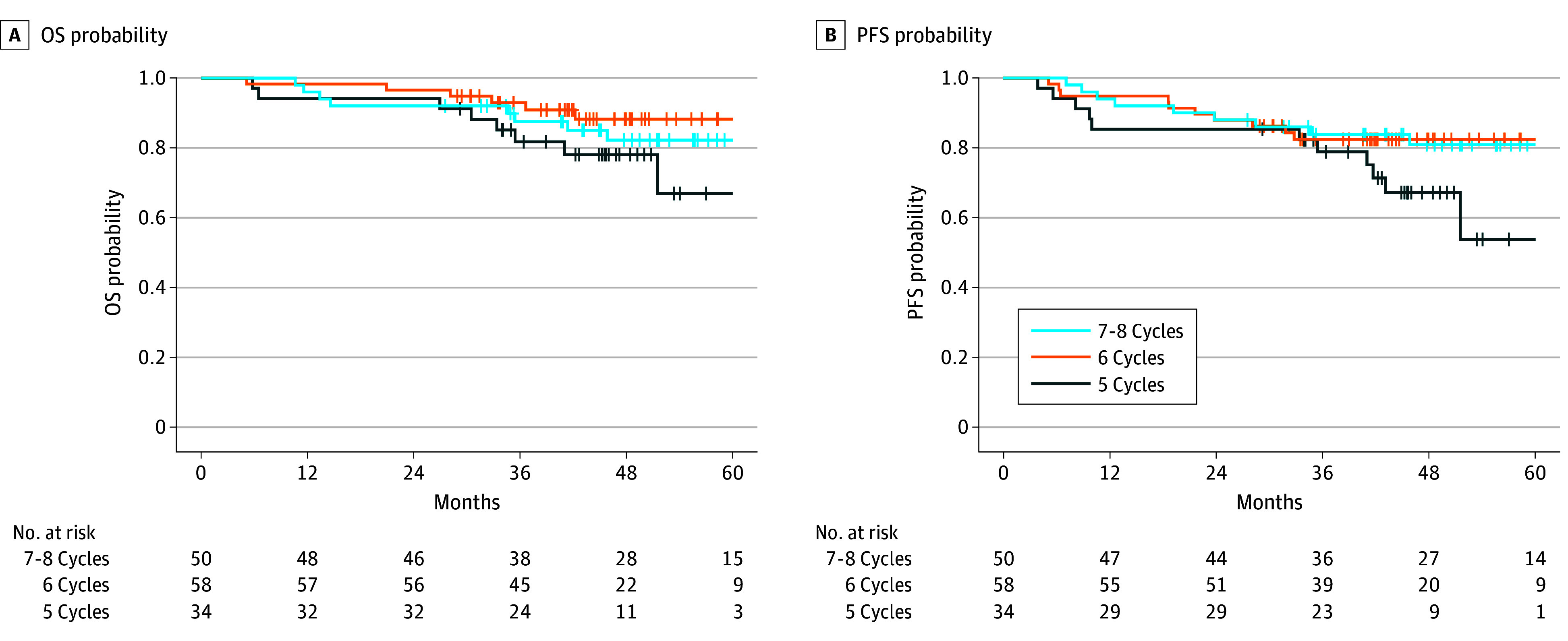
Kaplan-Meier Plots for Survival Outcomes Comparing Patients Who Received 5, 6, and 7 to 8 Weekly Cisplatin Cycles OS indicates overall survival; PFS, progression-free survival.

**Figure 2.**
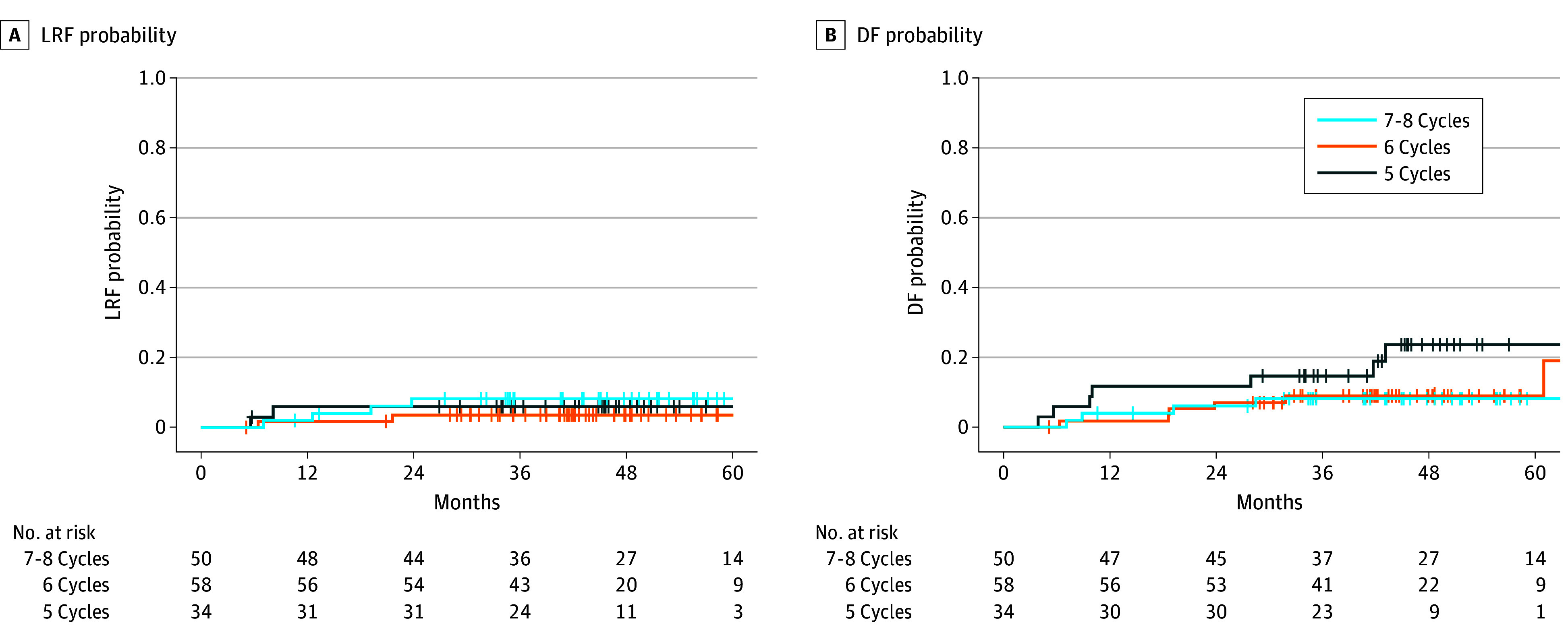
Cumulative Incidence Plots for Cancer Control Outcomes Comparing Patients Who Received 5, 6, and 7 to 8 Weekly Cisplatin Cycles DF indicates distant failure; LRF, locoregional failure.

On Cox and Fine-Gray MVA for the entire cohort, those who missed weekly cisplatin cycles had worse OS (for every missing cisplatin cycle, adjusted hazard ratio [aHR], 2.22; 95% CI, 1.19-4.17; *P* = .01) and PFS (aHR, 1.83; 95% CI, 1.06-3.15; *P* = .03) than those who received 7 to 8 cycles (Table 3 and Figure 1). For those receiving 7 to 8 cycles, the median (IQR) OS was 51.0 (40.7-61.8) months, and the 3-year OS was 88% (95% CI, 79%-97%). For those receiving 6 cycles, the median (IQR) OS was 43.6 (38.5-53.4) months, and the 3-year OS was 93% (95% CI, 86%-100%). For those receiving 5 cycles, the median (IQR) OS was 45.4 (35.1-49.8) months, and the 3-year OS was 82% (95% CI, 69%-96%). For those receiving 7 to 8 cycles, the median (IQR) PFS was 50.0 (35.0-61.1) months, and the 3-year PFS was 84% (95% CI, 74%-95%). For those receiving 6 cycles, the median (IQR) PFS was 42.8 (33.0-53.4) months, and the 3-year PFS was 82% (95% CI, 73%-93%). For those receiving 5 cycles, the median (IQR) PFS was 42.9 (34.3-48.1) months, and the 3-year PFS was 79% (95% CI, 66%-94%). Cancer control outcomes were comparable between these groups (LRF: aHR, 0.53; 95% CI, 0.15-1.93; *P* = .34; DF: aHR, 1.51; 95% CI, 0.60-3.82; *P* = .38) (Table 3 and Figure 2).

**Table 3.** Multivariable Analyses for Survival and Cancer Control Outcomes

Variable	Overall survival		Progression-free survival		Locoregional failure		Distant failure	
	aHR (95% CI)	*P* value	aHR (95% CI)	*P* value	aHR (95% CI)	*P* value	aHR (95% CI)	*P* value
Cisplatin cycle, for every missing cisplatin cycle	2.22 (1.19-4.17)	.01	1.83 (1.06-3.15)	.03	0.53 (0.15-1.93)	.34	1.51 (0.60-3.82)	.38
Age, y								
<65	1 [Reference]	NA	1 [Reference]	NA	1 [Reference]	NA	1 [Reference]	NA
≥65	0.82 (0.27-2.48)	.72	1.17 (0.47-2.88)	.74	<0.001 (<0.001-<0.001)	<.001	4.10 (0.97-17.28)	.06
Gender								
Female	1 [Reference]	NA	1 [Reference]	NA	1 [Reference]	NA	1 [Reference]	NA
Male	0.62 (0.22-1.75)	.36	1.11 (0.38-3.29)	.84	1.70 (0.45-6.46)	.43	5.16 (0.37-72.44)	.22
Race								
White	1 [Reference]	NA	1 [Reference]	NA	1 [Reference]	NA	1 [Reference]	NA
Other^a^	1.62 (0.38-6.93)	.52	2.23 (0.61-8.21)	.23	<0.001 (<0.001-<0.001)	<.001	19.0 (3.56-101.88)	<.001
Smoking								
Never	1 [Reference]	NA	1 [Reference]	NA	1 [Reference]	NA	1 [Reference]	NA
Former	3.23 (0.94-11.16)	.06	2.61 (0.80-8.51)	.11	1.55 × 10^12^ (2.09 × 10^10^-1.16 × 10^14^)	<.001	1.20 (0.22-6.65)	.84
Current	1.65 (0.43-6.26)	.47	2.71 (0.79-9.27)	.11	6.59 × 10^12^ (3.70 × 10^10^-1.18 × 10^15^)	<.001	1.77 (0.35-9.07)	.49
Not available	0.71 (0.05-9.39)	.80	6.15 (0.66-57.51)	.11	1.08 × 10^7^ (1.52 × 10^5^-7.75 × 10^8^)	<.001	127.63 (1.66-9822.29)	.03
Primary site								
Oropharynx	1 [Reference]	NA	1 [Reference]	NA	1 [Reference]	NA	1 [Reference]	NA
Larynx	3.65 (0.42-31.44)	.24	2.07 (0.34-12.65)	.43	<0.001 (<0.001-<0.001)	<.001	0.11 (0.004-2.60)	.17
Other	3.62 (0.59-22.31)	.17	2.31 (0.45-11.83)	.32	0.15 (0.01-1.74)	.13	0.99 (0.14-7.19)	.99
Body mass index^b^								
Normal (18.5-24.9)	1 [Reference]	NA	1 [Reference]	NA	1 [Reference]	NA	1 [Reference]	NA
Underweight (<18.5)	21.59 (3.64-128.21)	<.001	9.86 (2.10-46.36)	.004	126 (0.28-5.67 × 10^4^)	.12	25.74 (4.73-140.14)	<.001
Overweight (25.0-29.9)	0.06 (0.02-0.24)	<.001	0.27 (0.10-0.76)	.01	0.96 (0.45-20.3)	.98	0.10 (0.02-0.60)	.01
Obese (≥30.0)	0.10 (0.03-0.34)	<.001	0.22 (0.07-0.64)	.006	0.30 (0.02-4.21)	.37	0.07 (0.01-0.45)	.005
Eastern Cooperative Oncology Group performance status								
0	1 [Reference]	NA	1 [Reference]	NA	1 [Reference]	NA	1 [Reference]	NA
>0	5.76 (2.36-14.03)	<.001	2.40 (1.02-5.62)	.04	0.73 (0.05-10.2)	.82	1.21 (0.10-14.29)	.88
T category								
1-2	1 [Reference]	NA	1 [Reference]	NA	1 [Reference]	NA	1 [Reference]	NA
3-4	1.33 (0.49-3.61)	.57	1.21 (0.53-2.78)	.65	0.61 (0.09-4.20)	.61	1.36 (0.32-5.76)	.67
N category								
0-1	1 [Reference]	NA	1 [Reference]	NA	1 [Reference]	NA	1 [Reference]	NA
2-3	1.44 (0.54-3.88)	.47	1.60 (0.57-4.44)	.37	9.76 (0.49-193)	.13	20.16 (3.00-135.61)	.002
Human papillomavirus								
Negative	1 [Reference]	NA	1 [Reference]	NA	1 [Reference]	NA	1 [Reference]	NA
Positive	3.92 (0.49-31.65)	.20	3.02 (0.47-19.34)	.24	<0.001 (<0.001-<0.001)	<.001	1.58 (0.14-18.10)	.71

Abbreviations: aHR, adjusted hazard ratio; NA, not applicable.

^a^ Other includes but is not limited to African American, American Indian or Alaska Native, Asian, Hispanic, and those with unknown race or who declined to answer. These racial categories were grouped together because of the small sample sizes.

^b^ Body mass index is calculated as weight in kilograms divided by height in meters squared.

On our pairwise comparisons, compared with those receiving 7 to 8 cycles, patients receiving 6 cycles had comparable OS (aHR, 0.45; 95% CI, 0.11-1.82; *P* = .37), whereas those receiving 5 cycles had worse OS (aHR, 5.12; 95% CI, 1.35-19.45; *P* = .01). Compared with those receiving 6 cycles, patients receiving 5 cycles also had worse OS (aHR, 11.42; 95% CI, 2.57-50.63; *P* < .001). Compared with those receiving 7 to 8 cycles, patients receiving 5 (aHR, 3.23; 95% CI, 0.99-10.60; *P* = .05) and 6 (aHR, 0.86; 95% CI, 0.28-2.66; *P* = .95) cycles both had comparable PFS. However, compared with those receiving 6 cycles, patients receiving 5 cycles had worse PFS (aHR, 3.77; 95% CI, 1.23-11.58; *P* = .02).

On our exploratory subgroup analysis, among those with p16-negative tumors (32 patients [22.5%]), patients who missed weekly cisplatin cycles had worse OS (aHR, 11.34, 1.51-84.94; *P* = .02) than those treated with 7 to 8 cycles, but PFS was comparable (aHR, 3.70; 95% CI, 0.31-43.52; *P* = .30). However, among those with p16-positive tumors (110 patients [77.5%]), there were no statistically significant differences in OS (aHR, 1.21; 95% CI, 0.47-3.14; *P* = .69) and PFS (aHR, 1.32; 95% CI, 0.65-2.68; *P* = .45) between those who missed weekly cisplatin cycles vs patients who received 7 to 8 cycles.

## Discussion

To our knowledge, this is the largest cohort study of US patients with head and neck cancer who underwent definitive chemoradiation with weekly cisplatin reporting outcomes stratified by p16 status. These findings suggest that nearly one-quarter of our patients missed several cycles of weekly cisplatin and that cytopenia represented the most common reason for cisplatin interruption. The findings also suggest that patients who missed weekly cisplatin cycles and only received 5 total cycles had lower OS than those who received 7 to 8 cycles, although oncologic outcomes were comparable. This finding may be potentially associated with p16-negative tumors, although the sample size of patients with such tumors was small. Survival outcomes for patients with p16-positive tumors were not associated with the number of cisplatin cycles.

In our study, 18 patients (11.3%) who underwent fewer than 5 weekly cisplatin cycles were excluded for analysis given its small sample size. Such a proportion of patients was favorable and consistent with 13% to 32% of patients receiving fewer than 5 weekly cisplatin cycles reported in other studies.^5,6,7^ Among those who received 5 or more weekly cisplatin cycles in our study, nearly one-quarter of patients missed several planned cisplatin cycles with cytopenia as the most common reason. Such a finding was consistent with an Australian study^7^ reporting myelosuppression as the most common reason for cisplatin interruption. Reasons for cisplatin interruption were not well reported among prospective studies for comparison. For instance, RTOG 0129, RTOG 1016, and a phase 3 trial from India reported unspecified toxicities as one of reasons for cisplatin interruption.^3,4,8^

Our study suggested that those who missed weekly cisplatin cycles had poor survival outcomes with comparable oncologic outcomes. Such findings may be in part explained by substantial symptom burden, including failure to thrive with worsening performance status and frequent hospitalization for infection workup that led to cisplatin interruption. For instance, a prior study^9^ showed that dehydration and fever were the most frequent reasons for hospitalization and that hospitalization was associated with poor OS outcomes among patients with head and neck cancer. Symptom monitoring has been shown to improve adherence to chemotherapy^10^ and even OS among patients with metastatic cancer.^11^ A similar trial is currently ongoing among patients with head and neck cancer receiving chemoradiation.^12^

Our finding of poor survival outcomes for those with p16-negative tumors who missed weekly cisplatin cycles may be hypothesis-generating and needs to be further validated in future studies. In addition to symptom burden as described previously, a potential rationale for this finding is that adherence to systemic therapy may be more important among patients with more aggressive tumor biology. For instance, the majority of patients in both Australian and Indian studies had laryngeal and hypopharyngeal cancer, and poor adherence to weekly cisplatin was associated with worse survival outcomes.^7,13^ In contrast, p16-positive oropharyngeal cancer is known to have more favorable prognosis,^14^ and there was no statistically significant benefit in outcomes for receiving sixth or subsequent weekly cisplatin cycles in our subgroup with p16-positive tumors. Such findings were consistent with RTOG 0129, suggesting the lack of statistically significant benefits in receiving third cisplatin cycle.^3^

Given a lack of patients with p16-positive oropharyngeal cancer in recent phase 3 trials of weekly cisplatin,^4,5^ results from the currently ongoing NRG HN009 trial are eagerly awaited.^15^ Although its protocol did not specify the minimum number of weekly cisplatin cycles to be delivered for protocol compliance, its results may shed light on outcomes among patients with different levels of adherence to weekly cisplatin.

### Limitations

Our limitations include the retrospective nature of the study. Although all patients started with 40 mg/m^2^ for weekly cisplatin, doses might have been decreased in certain circumstances among select patients for better tolerability during subsequent cycles. Such dose adjustments were unavailable for review in our database, and not all patients who received 5 weekly cisplatin cycles had a cumulative dose of 200 mg/m^2^. However, despite such limitations, oncologic outcomes such as LRF and DF were not statistically significantly different for our entire cohort as well as subgroups. Other clinically relevant variables, such as medical comorbidities and toxicity profiles, were unavailable for analysis. As a result, despite the use of MVA to adjust for baseline characteristics, unmeasured confounders may exist. Although differences in survival outcomes for those with p16-negative tumors could be explained, in part, by differences in overall health status as suggested by different chemotherapy cycles being tolerated, our analyses adjusted for baseline ECOG PS and our entire patient cohort had comparable ECOG PS regardless of the number of chemotherapy cycles. In addition, differences in survival outcomes were not observed among those with p16-positive tumors.

## Conclusion

In our study, those who missed weekly cisplatin cycles had lower survival outcomes than others who received 7 to 8 cycles, although oncologic outcomes were comparable. Such findings may be potentially associated with p16-negative tumors, although this subgroup had a small sample size. Cytopenia represented the most common reason for missing cisplatin cycles.

## Notes

Supplement.
Data Sharing Statement
