# Supplementary material for: Weekly Cisplatin Cycles and Outcomes for Chemoradiation in Head and Neck Cancer
Source: JAMA Netw Open. 2024 Dec 9;7(12):e2450272. doi: 10.1001/jamanetworkopen.2024.50272 (PMC11629129; doi:10.1001/jamanetworkopen.2024.50272)
Supplement: Supplement. — Data Sharing Statement [file jamanetwopen-e2450272-s001.pdf]

Data Sharing Statement

Ma. Weekly Cisplatin Cycles and Outcomes for Chemoradiation in Head and Neck Cancer. *JAMA Netw Open*. Published December 11, 2024. doi:10.1001/jamanetworkopen.2024.50272

Data
Data available: No
